# Women Surgeons’ Experiences of Interprofessional Workplace Conflict

**DOI:** 10.1001/jamanetworkopen.2020.19843

**Published:** 2020-10-08

**Authors:** Lesly A. Dossett, C. Ann Vitous, Kerry Lindquist, Reshma Jagsi, Dana A. Telem

**Affiliations:** 1 Center for Health Outcomes and Policy, University of Michigan Institute for Health Policy and Innovation, Ann Arbor; 2 Department of Surgery, Michigan Medicine, Ann Arbor; 3 University of Michigan Medical School, Ann Arbor; 4 Department of Radiation Oncology, University of Michigan, Ann Arbor

## Abstract

**Importance:**

Gender differences in interprofessional conflict may exist and precipitate differential achievement, wellness, and attrition in medicine.

**Objective:**

Although substantial attention and research has been directed toward improving gender equity in surgery and addressing overall physician wellness, research on the role of interprofessional conflict has been limited. The objective of this study was to understand scenarios driving interprofessional conflict involving women surgeons, the implications of the conflict on personal, professional, and patient outcomes, and how women surgeons navigate conflict adjudication.

**Design, Setting, and Participants:**

A qualitative approach was used to explore the nature, implications, and ways of navigating interprofessional workplace conflict experienced by women surgeons. The setting was a national sample of US women surgeons. Purposive and snowball sampling were used to recruit women surgeons in training or practice from annual surgical society meetings. Participants were eligible if they were currently in a surgical training program or surgical practice. Nearly all participants had experienced at least 1 workplace conflict with a nonphysician staff member resulting in a formal write-up.

**Exposures:**

A workplace conflict was defined as any conflict resulting in the nonphysician staff member taking action such as confronting the woman surgeon, reporting the event to supervisors, or filing a formal report.

**Main Outcomes and Measures:**

Interviews were conducted between February 19, 2019, and June 21, 2019. Recordings were transcribed and deidentified. Inductive thematic analysis was used to examine data in relation to the research questions.

**Results:**

Thirty US women surgeons (8 [27%] age 25-34 years, 16 [53%] age 35-44 years, 5 [17%] age 45-54 years, and 1 [3%] age 55-64 years) of varying surgical specialties were interviewed. Conflicts were often reported as due to a breakdown in communication or from performance-related disputes. Participants perceived personal and professional implications including self-doubt, depression, frustration, anxiety, loss of sleep, reputational harms, and delays to advancement. Participants also described potential patient safety implications primarily due to decreased communication resulting from some surgeons being hesitant to engage in subsequent interactions. Participants described a variety of navigation strategies including relationship management, rapport building, and social capital. The success of these processes tended to vary by individual circumstances, including the details of the conflict, practice setting, level of support of leadership, and individual personality of the surgeon.

**Conclusions and Relevance:**

This qualitative study highlights women surgeons’ experiences with interprofessional workplace conflict. Interprofessional culture building, broader dissemination of implicit bias training, and transparent and equitable adjudication systems are potential strategies for avoiding or mitigating the implications of these conflicts.

## Key Points

**Question:**

What is the nature of interprofessional workplace conflicts experienced by women surgeons?

**Findings:**

The results of this qualitative study of 30 US women surgeons suggest that the circumstances leading to interprofessional conflicts were primarily associated with breakdowns in communication, performance-related disputes, or breaches of protocols. Women surgeons perceived (1) a double standard related to these conflicts, (2) the expectation that they conform to gender over professional norms, and (3) that these conflicts have negative consequences on their personal well-being and professional reputation.

**Meaning:**

These data support the need for systematic changes to prevent interprofessional workplace conflict and to ensure more equitable adjudication when conflicts arise.

## Teaser

This qualitative study uses data from interviews via email and national surgical societies to investigate interprofessional workplace conflicts experienced by women surgeons.

## Introduction

Women surgeons experience less achievement, are more dissatisfied, and have higher levels of burnout compared with their male colleagues.^1,2,3,4^ Substantial work has focused on improving gender-based achievement through increased implicit bias training, mentorship and sponsorship, and work-life integration policies.^2,5,6,7,8^ Despite this work, little has been done to explore the role that interprofessional relationships and conflict may have in gender-based achievement and satisfaction gaps for women surgeons.

Interprofessional teamwork is critical to success in medicine, particularly in intense work environments such as the operating room, trauma resuscitation bay, and critical care units.^9,10^ Interprofessional conflict is known to contribute to workplace dissatisfaction and stress, and existing data suggest women are more likely to experience these conflicts.^11^ For example, surgical technologists file complaints against women more than men and exhibit sex-based discrimination.^12^ Moreover, a study of operating room behaviors demonstrated complex differences related not simply to the sex of the attending surgeon but also the sex of others present, including greater cooperation when the attending surgeon’s sex differed from the majority of other team members. These gender-based differences in interprofessional conflict in surgical settings may partially explain why women surgeons experience a higher stress index.^13,14^

The purpose of this study is to understand interprofessional conflict involving women surgeons for the purpose of identifying strategies to prevent or more quickly resolve these conflicts. Our specific questions were: (1) what are the circumstances leading to interprofessional conflicts involving women surgeons, (2) what are the personal, professional, and patient safety implications of these conflicts, and (3) how do women surgeons navigate these conflicts and the resulting adjudication process?

## Methods

### Study Design and Setting

In order to broadly explore interprofessional conflicts experienced by women surgeons, we selected a qualitative descriptive study design utilizing semistructured interviews with a national sample of US women surgeons. The interviews sought to obtain a broad understanding of the nature of interprofessional conflicts experienced by women surgeons, the personal and professional implications of those conflicts, and strategies for navigating these conflicts. This study was approved by the University of Michigan Institutional Review Board and is reported according to the Consolidated Criteria for Reporting Qualitative Research (COREQ) reporting guideline (eAppendixes 1-3 in the Supplement).^15^

### Interview Participants

Participants were recruited by email (eAppendixes 1-3 in the Supplement) and in person at the annual meetings of national surgical societies. Purposive and snowball sampling were used to ensure participants were diverse with respect to age, surgical specialty, and years in practice (Table 1). Women surgeons were eligible if they were currently in a surgical training program or surgical practice. Nearly all participants had experienced at least 1 workplace conflict with a nonphysician staff member resulting in a formal write-up. Participants were not compensated for their participation. We continued to interview until thematic saturation was reached (ie, new themes emerged infrequently, and the code definitions remained stable), resulting in a total of 30 interviews.^16^

**Table 1.** Participant Demographic, Training, and Practice Characteristics

Category	No. (%)
Total No. of participants	30
Age, y	
25-34	8 (27)
35-44	16 (53)
45-54	5 (17)
55-64	1 (3)
Time since training, y	
0-5	13 (44)
6-10	3 (10)
11-15	5 (17)
16-20	1 (3)
>20	1 (3)
Current trainee	7 (23)
No. of positions held	
1	14 (47)
2	9 (30)
Current trainee	7 (23)
Time in current position, y	
0-5	20 (67)
6-10	6 (20)
11-15	4 (13)
Specialty	
Bariatric	1 (3)
Breast	3 (10)
Cardiac	1 (3)
Colorectal	2 (7)
General	10 (33)
Hepatobiliary	1 (3)
Otolaryngology	1 (3)
Pediatric	2 (7)
Plastics	4 (13)
Surgical oncology	1 (3)
Trauma	2 (7)
Urogynecology	1 (3)
Vascular	1 (3)

### Interview Procedures

All participants received a written informed consent statement and verbally consented before their interview. Participants agreed to having their responses published, and quotations were shared with all participants to ensure accuracy. Interviews were conducted in person or over the phone between February 19 to June 21, 2019, by a coinvestigator (C.A.V.), a woman anthropologist with significant expertise in qualitative interviewing. The interviewer had no previous relationship established with participants before the interview. An interview topic guide (eAppendix 2 in the Supplement) was designed to explore experiences of workplace conflict resulting in reporting action by a nonphysician (eg, nurse, scrub tech, or circulator). A workplace conflict was defined as any event or conflict leading the staff member to take action such as directly confronting the woman surgeon, reporting the event to a supervisor, or filing a formal report. The domains included in the guide were workplace culture, descriptions of workplace conflict, implications of workplace conflict, and how participants would have preferred conflicts be handled. Several iterations of the interview guide were generated based on content validity, face validity, ability of participants to interpret essential information, and ability to complete the interview within the anticipated time. Two pilot interviews were completed with subsequent slight modification to the interview guide. The interviews were digitally recorded and lasted an average of 32 minutes (range, 13-53 minutes). Observations about each interview (ie, field notes) were documented afterward.

### Analysis

Audiotapes were transcribed verbatim and deidentified. Once data were collected, we began an iterative process of analyzing the data using inductive thematic analysis. The initial codebook was created by having 2 team members (C.A.V. and K.L.) read the transcripts and collate ideas, mapping them to a coding schema. Once the initial codebook was agreed upon, each transcript was then coded using NVivo software, version 12.5.0 (QSR International) by 2 members of the research team (C.A.V. and K.L.), blinded to each other’s work. In areas where disagreement was found, consensus was met between C.A.V. and K.L. Once all data were coded, the entire research team met to reach consensus on the most salient themes.^16^ Validity was established through investigator triangulation and member checking, which was accomplished by sharing study findings with participants to ensure the findings represented their viewpoints.^13^

## Results

We interviewed 30 women surgeons (8 [27%] age 25-34 years, 16 [53%] age 35-44 years, 5 [17%] age 45-54 years, and 1 [3%] age 55-64 years) from the United States from a variety of surgical specialties (Table 1). The majority of participants were younger than 45 years and had been in practice for less than 10 years, consistent with the distribution of age and experience level among women surgeons in the United States.^17^ Inductive thematic analysis of the narratives of the women surgeons in this study revealed 3 major themes in relation to circumstances of interprofessional workplace conflict:

1. Circumstances of reported conflicts: the context surrounding the conflict events (eg, performance-related issues, lack of professionalism, breaches of policies or protocol)
2. Implications of conflict: the perceived effects of the conflict events on the women surgeons (eg, personal, professional, patient outcomes)
3. Strategies for navigating conflict: the approaches described by women surgeons as being helpful in navigating interprofessional conflicts (eg, relationship management, rapport building, social capital)

### Circumstances of Reported Conflicts

Circumstances leading to reported interprofessional conflicts were described by the participants as due to (1) the surgeon’s response to perceived performance-related issues on behalf of the staff member, (2) the interprofessional staff perceiving the women surgeons as unprofessional, (3) breaches of protocols, or (4) a combination of these circumstances (Table 2). In addition, participants described the role of double standards and difficulty in taking orders from a woman as factors mediating these circumstances.

**Table 2.** Circumstances of Reported Conflicts

Theme	Exemplary quote
Performance-related issues	“I was calling the emergency room, asking them to get her in, to get her worked up. And it was too slow, and I asked to talk to the nurse in charge. And I was like, ‘Please,’ you know? And I was very straightforward and demanding to her. And then I was written up for that, mostly because I think she thought her job was threatened.”
	“And they do things like give steroids for sepsis right off the bat, which is not within the guidelines of the Surviving Sepsis Campaign. So I spend a lot of time in the ICU [intensive care unit] explaining myself, because the nurses question us quite a bit.”
	“The circulating nurse and the scrub nurse in the room, they didn't have the equipment we needed for the operation we planned to do. We were not doing anything out of the ordinary. It was a very standard kind of operation. And we needed standard things that were on my list. And literally within 15 minutes of getting into the room, I said, ‘Hi, how are you doing?’ And then there was initial conflict about, ‘Well, we don't have what you need.’ And I kind of felt the vibe in the room as being pretty confrontational.”
Unprofessional behavior	“I got pulled into the charge nurse’s office and she said, ‘The nurses don’t like the way you’re interacting with them. They don’t think you are being helpful. They don’t think you are playing nice.’”
	“It's always because you hurt someone's feelings, and it's never because you threw something…It's usually because we just weren't nice [enough], or we weren't holding their hand, or we didn't allow them to make a mistake.”
Breaches of institutional policies or protocols	“I wore the long [disposable gown] in the operating room because I’m petite, and I get cold…And I went probably about 6 months doing that, and no one said anything to me. And then all of a sudden, I was walking to the OR [operating room], and a nurse kind of came up and accosted me. It was like, ‘You can’t wear that.’ And I was like, ‘Oh, why not?’ And she’s like, ‘It’s policy. You can’t wear the long ones in there. It has to be the short one.’”
Mediating circumstances	
Taking orders from a woman	“I think there is an inherent tension between women physicians and women nurses…I don’t know what it’s about, if it’s about like, they think we think we're better than them? They kind of don’t want to help you the way they would help a man.”
	“Women don't like to take orders from other women. They’re perfectly okay taking any kind of order from a man, but they really have a hard time taking orders from another woman.”
	“I find females against females tend to harbor some sort of need to feel one-upped, or need to assert their position—like the scrub nurse needs to assert this is her space with another female surgeon. And you know, a female surgeon, if she was to do it or respond in kind to a way than a male might, would easily be perceived as a bitch.”
	“I think it's very hard for women in nonphysician roles to accept a woman in a position of power, or so-called power, since sometimes I don't feel very powerful as a [specialty] surgeon. [They] accept orders or behavior easier from a man. I believe that.”
Double standards	“Guys tend to do a lot of ranting and yelling and throwing things, and it's just like completely ignored. I don't get that. I know some attendings here that got into a fist fight in the OR [operating room] once, and I don't think anybody got sent to a life coach, right? It's just like stunning to me.”
	“I was told I was breaking the rules, and yet nobody could actually tell me what the rules were because it seemed to me that this other person had booked a case into the emergency-use OR [operating room] and was sitting at [*sic*] was also breaking the rules. I was like, well, if nobody else is playing by the rules, then why do I have to play by the rules? It also happens to be a male physician.”
	“I think for women, if you get upset or react to a situation, you’re considered emotional or reactionary, whereas if a man does it, they’re being assertive or advocating for their patients.”
	“Women in surgery, I think, get held to a different, sometimes unfair standard.”
	“And then I was stunned at the end of the meeting when he told me he understood that, yes, sometimes men and women who may say the exact same thing in the exact same tone, we may be perceived differently. And I was very happy to hear him acknowledge that. But in the very next breath, he said, ‘Maybe you would like to pursue some coaching to help with the way people perceive you?’”

Performance-related issues included a failure of the staff member to obtain proper equipment for cases, schedule or prioritize clinical work, carry out clinical care, or communicate about a patient’s status. Participants described how when they attempted to correct these issues with the involved staff, they were regularly perceived as being unprofessional. Often, these events or complaints were escalated to leadership, with the surgeon having limited knowledge of whom, or in some instances what specific event, led to the write-up. In the majority of cases, the conflict occurred when the women surgeons addressed the performance issue in a way that the staff perceived as unprofessional behavior. These responses were characterized as “sarcastic” or “mean” by the staff member (occasionally the women surgeons agreed with these characterizations). Notably, the women surgeons believed their responses were in line with those they had witnessed from male mentors or colleagues.

Other conflicts resulted from breaches of institutional policies or protocols by the women surgeons (policies for booking urgent cases, wearing nail polish, wearing incorrect operating room attire, etc), most often occurring in the operating room suite. Many of these conflicts related to nursing-derived policies the women surgeons believed were not supported by evidence (eg, rules against the use of cloth scrub caps or scrub jackets). Participants further described a double standard in enforcing a policy when a woman surgeon was involved while allowing male surgeons to breach the same policy.

Participants described events involving other women interprofessional staff and often speculated that these staff members are unaccustomed to or uncomfortable with taking direction or being led by other women. For many participants, these processes were difficult to tease out against other aspects possibly at play—such as tension between nursing and surgical culture, being a person of color, or the particular place in their career (ie, early in their career). The participants further believed that this led to interprofessional staff having a double standard with regard to how behavior was accepted, reported, or adjudicated. This occurred at the level in which the event was happening as well as how leadership responded to it. Importantly, in many examples, the reporting came as a surprise to the women surgeons, as they had not perceived a significant conflict with the interprofessional staff member. Participants often further clarified that they did not perceive a malicious intent on the part of the staff but rather acknowledged many of these interactions reflected the bias and gender scripts present in society at large.

### Implications of Conflict

Participants described a number of implications arising from interprofessional workplace conflict, falling under the domains of personal, professional, and patient outcomes (Table 3). Personal implications included both emotional and physical components. From an emotional standpoint, participants described feelings of self-doubt, depression, frustration, devastation, humiliation, and anxiety. Though not as common, participants asserted a number of physical symptoms including gastrointestinal distress, loss of appetite, insomnia, burnout, and exhaustion. Participants described how they perceived these responses to affect their mood and influenced the way they felt both in the hospital and at home. There was also variation in how these responses were experienced, in both duration and intensity.

**Table 3.** Implications of Conflict

Theme	Subtheme	Exemplary quote
Personal	Emotional	“It was really stressful because I had never been written up before. I'm one of those people that [people] like saying things about me, like making me sound like lazy or a bully. It hurts because, you know, I never want anyone to think I'm a workplace bully. I pride myself on working really hard with people. And so it just really made me feel very stressed, and I didn't know what was going to happen as well.”
		“I feel like I’m trying really hard to take care of patients and operate and work long hours, and then to have these incidents [that] actually don’t matter and don’t affect patient care—it’s kind of draining and demoralizing…”
		“At some point, it does undermine your mental and physical well-being, being anxious on a daily basis of what the heck is coming next? What are they going to do now?”
		“But, yeah, I mean, certainly it affects me, because again, I'm using a lot of emotional resources to try to figure out if it’s me as a person. Am I doing something wrong to elicit these responses, or is it that they really, truly are not wanting to take orders from a person who’s younger than them, a person who is not a man, and a person who is a different race than them?”
	Physical	“At baseline, I tend to get pretty severe gastrointestinal distress when I am stressed, so I wound up with a lot of GI [gastrointestinal] upset, which did affect my diet.”
		“I have eczema, which is stress-related, and it flares. And I'll be honest, when I have a lot of events around my leadership, then it does flare.”
Professional	Reputation	“It's like, where did this go, and how does that affect your reputation, when really, you were not at fault and did not do what you were accused of doing.”
		“It’s way easier to put out a bad rumor than it is to pull it back or to mediate it. I think that was a major factor in me developing a reputation of being rude or overconfident or any of the things I was labeled [as being].”
		“I think a lot of the people in my department had a negative opinion of me after this. They just viewed me as somebody who was hard to work with and I had problems communicating.”
	Career trajectory	“It has delayed my promotion. I'm 12 years in now and that delayed me [from] even being put up for a promotion.”
		“I have a very low satisfaction with my work environment and my colleagues. I’m probably in transition out of this job but also a little bit, eyes wide open, [understanding] there’s not a nirvana out there. But maybe being a little bit more thoughtful the next time around, I will look at a group where there are more women and maybe more mid-career or senior women.”
		“I couldn't advocate for my fellow residents while not seeming overconfident, so I withdrew from a leadership position. I avoided any interaction even with my fellow residents and particularly with attendings that [*sic*] were not like necessary. I just did what I could to get through it.”
Patient outcomes		“So when I walked into the OR [operating room] and I was ready to start, I said ‘Should we do a timeout?’ because when I come in as a second surgeon, we do a second timeout. And the nurse said ‘Well we already did the timeout.’ And I said ‘Yes, but I wasn’t here and I just want to make sure we’re doing the right thing.’ And she’s like, ‘Well, if [you] would calm down your hormones’…and I didn’t even know how to respond to that. And I simply said, ‘I just want to make sure you’re following your policy, so you know what I’m doing. If you don’t want to, if that’s how it’s going to be here, that’s fine.’”
		“It can affect patient care because it makes you not want to go back to the scene of the crime, if you will. I was probably less interactive in trying to engage the team in conversations about patient care.”

Professional implications included perceived harms to professional reputation, a reluctance to pursue leadership positions, and a perceived detrimental effect on promotion and career trajectory. Because of the lack of transparency and consistency in how these events were recorded in employee files, participants described how the exact implications of these events were often difficult to assess. Although some participants were able to cite explicit examples, many others described the subtle ways in which they came to realize how both colleagues and mentors perceived them.

Implications for patient outcomes included nonphysician staff questioning guidelines or orders from women surgeons resulting in delays in care, as well as some surgeons avoiding interprofessional engagement regarding patient issues owing to fear of conflict. Specifically, participants described impaired collaboration with potential patient outcome implications after a conflict, manifest in their own avoidance of further interactions with the staff member or tense and ineffective communication. Although the participants did not describe specific negative patient outcomes, they often referenced the belief that patient outcomes were improved when interprofessional communication and collaboration were optimal.

### Strategies for Navigating Conflict

Participants described a variety of ways in which they attempted to navigate these conflicts. Domains included relationship management, rapport building, and building social capital (Table 4). Specifically, participants described what could be collectively considered the unwritten rules of being a woman surgeon. For example, one participant remarked:

**Table 4.** Strategies for Navigating Conflict

Theme	Exemplary quote
Relationship management	“The fact people had such bad reactions, or bad responses or bad interpretations of the interactions, was something I can at least try to control. So, whether or not I was right, I don't see that as actually mattering. I see it as I did not give the impression that I wanted to give, and therefore, the only thing I can intervene upon is myself.”
	“[I use] things like, ‘We’re on the same team,’ or ‘Can we talk about what your goals are,’ redirecting and bringing together conversations, or maybe aligning conversations, I’ve been able to use those techniques in most conflict situations.”
Rapport building	“The warnings I received from more senior women, was [*sic*] that the nurses weren't going to treat us as well and didn't listen to us and that to get anywhere, you had to be really social and friendly and become friends with everyone.”
	“I am someone who learned pretty quickly that as a woman, I needed to make friends with the nurses, and it was going to be harder for me to get them to do what I wanted. That was obvious to me from the get-go. And so, I felt it was an obligation to make, quote, unquote, friends with them in order to achieve what I needed to be done for patient care.”
	“If the male surgeons lose their mind, they just kind of duck and cover and go about their way without necessarily building that relationship more. I think being female gives me an advantage in those relationships in some ways. But then I think it also gives me a disadvantage when you really need to get things done. It works against you.”
Social support	“One of my female attendings reached out to me about it because I think she heard stuff through the grapevine and OR [operating room]…nurses talking about that and other interactions. And so, they’ve both reached out to me to figure out ways to make things better and, you know, tell me…the system.”
	“You just kind of deal with it on your own, or, you go have a drink with some of your colleagues who are experiencing the same things.”
	“I work with a lot of residents who are women, and we actually have like a women in surgery group we just started, in many ways to have a safe space to talk about these things, and [ask each other] ‘How did you deal with these things?’”

“I’ve had conversations with my female counterparts, and it’s always the same conversation. If you want something done, bring cookies. If you get mad, don’t show it. If you have conflict, address it head on, apologize. Even if you don’t think you’ve done anything wrong you’re still at fault. It’s just kind of a theme.”

From a relationship management perspective, participants discussed aspects such as personal accountability, gauging the emotional responses of others, and recalibrating their actions based on those responses. Often, participants described these processes as contributing to the emotional implications, as they were seen as a form of additional labor required of them. Participants also described the process of rapport building. Within this was the expectation to participate in events for nonphysician staff (ie, baby showers), to establish friendships, and the amount needed to invest in order to develop those relationships. For some, this process was natural and in line with how they would communicate with colleagues, but for others it felt contrived and was viewed as a form of performance needed to make things run smoother.

Participants also described the various ways in which they established social support in these spaces. In the absence of having leadership effectively manage these situations, women surgeons would find other forms of support to alleviate the burden. This support was found in both formal and informal spaces and most often involved commiserating over shared experiences. The success of these processes varied by individual circumstances, including the details of the conflict, practice setting, level of support of leadership, and individual personality of the surgeon.

## Discussion

This study has several key findings: (1) interprofessional conflict experienced by women surgeons was primarily due to a breakdown in communication, breaches of protocols, or the surgeons’ response to perceived performance-related issues; (2) women surgeons described various strategies for navigating these conflicts but ultimately expressed frustration regarding what they perceive as a double standard for behavior and a need to conform to gender over professional norms; and (3) women surgeons perceive these conflicts to have substantial personal and professional implications. These findings highlight opportunities for improvement in order to avoid conflict or more quickly and fairly adjudicate issues when they do arise.

Many conflicts described by participants arose from performance- or systems-based issues identified by the women surgeons that led them to address the performance gap in a way that was deemed unprofessional. The women surgeons largely perceived their actions as mirroring those of their male surgeon mentors or peers. The majority of conflicts were with other women, leading many participants to speculate conflict resulted from staff members not being comfortable with actions violating gender stereotypes, such as assertive direction or correction from another woman. This notion is supported by the literature where studies note the prescriptive nature of gender stereotypes and the discordance of expectations for conforming to these stereotypes among physicians and nurses.^18^ For example, whereas occupation is viewed as secondary to gender by female nurses, for female physicians, gender is viewed as secondary to occupation.^19^ This leads women physicians to feel as if they must conform to gender norms and be more polite with nurses than their male colleagues, even when certain clinical circumstances may call for highly agentic and stereotypical male behaviors.^20^ This perception is further supported by data demonstrating that expression of anger in the workplace may heighten status for men but may lead to backlash or lessen the status of women. Furthermore, women’s anger is more likely to be attributed to internal personality characteristics, whereas men’s anger is attributed to external circumstances or stressors.^21,22,23,24^

Although the number of women in surgery has increased in the past decade, the senior surgeons who train them remain predominately male. Some male surgeons continue to model behaviors projecting a patriarchal or dominant-subservient doctor-nurse relationship,^19^ which then leads to interprofessional conflict when expressed by women. Although a focus on professionalism, team building, a flattened hierarchy, and open communication^25^ has led to improvement in the accepted behavior in the operating room, there is undoubtedly room for continued improvement. Especially in training programs, hostile, uncivil, sarcastic, or bullying behavior should not be tolerated, whether from men or women. Second, interprofessional staff should consider their own explicit and implicit biases as a means of increasing awareness of differences in gender norms and expectations. Although implicit bias training has noted shortcomings, it is, at minimum, a means of addressing knowledge gaps that contribute to behavior.^26^ Finally, male leaders, when adjudicating conflict, should consider whether conflict with traditional gender schema could have perpetuated either the conflict or the conflict reporting. Just as a growing body of literature demonstrates female faculty are evaluated more harshly than their male peers,^27^ attention to whether a reported conflict is due to a particular behavior or a difference in reporting threshold should be considered.

These data also support a potential to prevent conflict through interprofessional team building and training. Despite the critical nature of teamwork in the operating room, surgeons rarely have significant input in choosing their team members, regular opportunities for performance evaluation, or regular opportunities for team-based training. In this way, the traditional nature of physician and nursing leadership silos may create obstacles to optimal teamwork and accountability. Many conflicts reported by the participants occurred early in the tenure of the women surgeons, and relationships often improved after several years, after the staff became more familiar with the women surgeons. Given that many conflicts were related to perceived performance gaps, strategies such as assigning high-performing staff members to new surgeons may reduce interprofessional conflict by reducing the performance-based gaps surgeons may encounter when in a new system. For example, assigning a lower-performing or new staff member to a new surgeon who is under considerable cognitive load due to inexperience or a new environment may put a situation particularly at risk for conflict. Furthermore, simulated operating room team training^28,29^may improve teamwork and communication and prevent conflict in high-stress environments where a majority of these conflicts occurred.

A final key finding is the implication that these conflicts have for wellness. Referring to prior data linking workplace conflict to insomnia, depression, and somatic symtpoms,^30,31,32^ these conflicts may have implications for retention, job satisfaction, and burnout for women surgeons. In order to be effective, comprehensive wellness efforts must aim to prevent burnout by addressing workplace conflict through the strategies noted previously or through more equitable adjudication processes.

### Limitations

Our study has several limitations. We relied on women surgeons to self-identify workplace conflicts and did not interview interprofessional staff regarding their experience of conflict with women surgeons. This possibly creates bias in the reported circumstances and only represents one side of the conflict. We also did not interview male surgeons regarding their experience with interprofessional conflict; it is possible some themes are not unique to conflict involving women surgeons. These areas are the focus of ongoing investigations. Finally, we did not deeply explore intersectionality with regard to race/ethnicity, experience level, or other factors.

## Conclusions

The results of this qualitative study of 30 US women surgeons suggest that the circumstances leading to interprofessional conflicts are primarily due to breakdowns in communication or performance-related disputes. Women surgeons perceive a double standard related to these conflicts and the expectation that they conform to gender over professional norms. Further research to elucidate drivers and outcomes of interprofessional conflict, including the role of gender, is necessary to inform policy and practice. Only by evidence-based intervention can patient care and professional wellness be optimized.

## Notes

Supplement. **eAppendix 1.** Recruitment Language **eAppendix 2.** Interview Guide **eAppendix 3.** Codebook Definitions
