# Supplementary material for: Women Surgeons’ Experiences of Interprofessional Workplace Conflict
Source: JAMA Netw Open. 2020 Oct 8;3(10):e2019843. doi: 10.1001/jamanetworkopen.2020.19843 (PMC7545297; doi:10.1001/jamanetworkopen.2020.19843)
Supplement: Supplement. — eAppendix 1. Recruitment Language eAppendix 2. Interview Guide eAppendix 3. Codebook Definitions [file jamanetwopen-e2019843-s001.pdf]

Supplementary Online Content

Dossett LA, Vitous CA, Lindquist K, Jagsi R, Telem DA. Women surgeons' experiences of interprofessional workplace conflict. *JAMA Netw Open*. 2020;3(10):e2019843. doi:10.1001/jamanetworkopen.2020.19843

eAppendix 1. Recruitment Language

eAppendix 2. Interview Guide

eAppendix 3. Codebook Definitions

This supplementary material has been provided by the authors to give readers additional information about their work.

© 2020 Dossett LA et al. *JAMA Network Open.*

eAppendix 1. Recruitment Language

We are reaching out to see if you would be interested in participating in our study addressing workplace conflict. In this study, we are seeking to understand circumstances and factors related to workplace conflict between women surgeons and other non-physician healthcare staff (nurses, therapist, pharmacist, etc.). We are particularly interested in events or conflicts that led the non-physician staff member to take action such as directly confronting the female surgeon, reporting the event to her supervisor, or filing a formal report with human resources or hospital administration. This interview will take approximately 45 minutes and is completely voluntary.

If you are interested in participating in this study, please respond to this message and we will set up a time for the interview (via phone or BlueJeans). Please let us know your availability and we will accommodate. We can also schedule in the evenings and early mornings if that works.

Thank you for considering to participate.

© 2020 Dossett LA et al. *JAMA Network Open.*

eAppendix 2. Interview Guide

Thank you for agreeing to patriciate in the interview. In this study, we are seeking to understand circumstances and factors related to workplace conflict between women surgeons and other non-physician healthcare staff (nurses, therapist, pharmacist, etc.). This interview will take approximately 45 minutes and is completely voluntary. All scenarios will be de-identified. You can choose to not answer any question or end the interview at any time. Do you have any questions?

1. First we would like to ask some basic demographic questions:
    a. What is your age?
    b. What is your specialty?
    c. Are you currently in training?
    d. How many years has it been since you finished training?
    e. How many positions have you held since finishing training?
    f. How long have you been in your current position?
    g. How many workplace conflicts have you had in your current position that resulted in a write-up?
    h. What about conflicts that did not result in a write up?
2. Briefly describe your workplace culture.
3. Briefly describe your overall job satisfaction.
4. Walk me through a time you experienced a workplace conflict with a non-physician healthcare worker (how it started, how it escalated, who was involved, where it occurred, etc.).
5. Briefly describe the other person/people that were involved in this event (age, gender, position, etc.)
6. What action did this person take?
7. Was there any formal disciplinary action taken against you? If so, what was the nature of the action? Were you made aware that a formal action would be reported? By whom?
8. How would you describe your relationship with the person before the event? What about after? (Prompt: did it change)
9. What impacts, if any, did this event have with other people in the department?
10. Were you offered or encouraged to participate in any personal or professional development programs as a result of this event? (For example, Anger Management or Professional Coaching). If so, please describe that experience.
11. What, if any, emotional impact did this event have on you? (For example, stress, anxiety, depression). How long did these issues last?
12. What, if any, physical impact did this event have on you? (For example, not sleeping, not eating or overeating, etc.). How long did these issues last?
13. What impact, if any, do you perceive this event had on your career trajectory? (For example, delayed promotion, removal of leadership positions, etc.)
14. In your opinion, was the action taken warranted? Outcome fair?
15. What do you think could have been improved in how this event was handled?
16. Were any acute stressors occurring at the time of the interaction (divorce, sleep deprivation, new baby, pregnancy, sick family member, etc.) If yes, how do you feel these factors influenced how things unfolded or were dealt with on your end?
17. In what ways, if any, did this experience influence how you approached conflict going forward?
18. Is there anything else about this event that you would like us to understand?
19. Are there any other events you would like to describe?
20. Do you have any other comments or questions?

© 2020 Dossett LA et al. *JAMA Network Open.*

eAppendix 3. Codebook Definitions

Workplace Culture: Descriptions of workplace culture defined as: "...the shared values, belief systems, attitudes and the set of assumptions that people in a workplace share. This is shaped by individual upbringing, social and cultural context."
Institutional Setting: Statements regarding the institutional setting (rural, urban, community, academic, geographic region, etc.)
Job Satisfaction: Description of job satisfaction. Include comments regarding technical skills as well as environment. Usually, respondents chose to rank on a scale from 1-10. Others ranked on a scale from "Not happy/satisfied" to "very happy/satisfied"
Case Scenarios:

- **Reason for Complaint:** Statements regarding what prompted complaint (scheduling issue, equipment, language, etc.)
- **Staff Involved:** Any demographic info on person/people involved in the event. Also code if unsure who made complaint.
- **Type of Complaint:** Direct confrontation, reporting to supervisor, filing a formal report. Include details of complaint.
- **Disciplinary Action Taken:** Include type of action taken, how it was communicated, by whom.
- **Action Warranted:** Statements relating to feelings on if the action taken by the non-physician staff was warranted
- **Opportunities to Address Complaint:** Statements regarding the opportunity to address complaint. Also code in instances where no opportunity was provided.
- **Interactions with Involved Staff (pre and post):** Statements regarding relationship with person both before and after the event (how relationship changed).
- **Support of Leadership:** Statements regarding the role or response from leadership**.** Also code for lack of support.
- **Impacts on Relationships in Department:** Statements regarding how these events impacted relationships between participant and other people in the department. Also apply in instances where there is a culture of gendered issues that impact relationships in department.
- **Impacts on Career Trajectory:** Statements regarding the role that these events had on the participant's career trajectory. Include comments on desire to change institutions. Also code for positive examples (when participants reflect on how professional development courses positively impacted career trajectory).
- **Impacts on Approach to Conflict:** Statements regarding how these events influenced general approach to handling conflict.
- **Personal/Professional Development:** Statements regarding being encouraged or offered to participate in any personal or professional development programs. Also include any informal advice given by colleagues.
- **Emotional Impacts:** Include feeling of being blindsided, anxiety, depression, burnout.
- **Physical Impacts:** Include exhaustion, not sleeping, overeating, not eating.
- **Acute Stressors:** Statements regarding other factors that were transpiring at time of interaction (divorce, sleep deprivation, new baby, pregnancy, sick family member, etc.).

Rapport Building: Phenomenon of women surgeons reporting the need to establish rapport/develop relationships with non-physician staff in order to have things run smoothly (befriending nurses outside of work, asking them questions about how their weekend was or how their children are doing). Also described as a form of negotiation between women surgeons and non-physician staff.

© 2020 Dossett LA et al. *JAMA Network Open.*

Double Standards: Statements relating to the different expectations and/or consequences for male versus female surgeons. Include statements regarding being accused of creating a hostile work environment, when they are simply asking for help, recruiting others for assistance, etc.
Respect: Statements regarding the perception that male surgeons are viewed as being more efficient, intelligent, capable, more valued, etc. Code for microaggressions (although maybe this should be separate?)
Opportunities: Statements regarding being passed up for leadership positions, advancements, etc. that are perceived to be at least in part due to gender.
Implicit Bias: Comments or statements regarding assumptions regarding gender (assumed to be support staff, being asked who will be operating, etc.). Include how participants are treated by patients.
Disconnect between Policy and Practice: Statements regarding the gap between policy and practice. For example, having diversity statements or recruitment efforts to bring women in but lacking practices that make it an inclusive space.
Language/Tone: Statements regarding women being labeled as mean, confrontational, bitchy, difficult. Also code for statements regarding the need for women surgeons to adjust language/tone when communicating with non-physician staff.
Nonverbal: Statements regarding how women surgeons are judged or penalized for facial expressions and physical appearance.
Examples of Egregious Acts (of men): Anecdotes or statements regarding behaviors of men surgeons that participants assert are normalized (believed to be acceptable).
Emotional Labor: Statements regarding the burden felt by participants of managing feelings and expressions (specifically as they differ from male colleagues). Include statements regarding the "unwritten rules" of being a women surgeon.
Impacts on Patient Outcomes: Statements regarding how events (or general culture) impact surgical outcomes.
Influence of Hierarchy: Statements regarding how experiences are different based on position (resident vs attending).
Timing/Generational Differences: Code when surgeons who have been practicing/out of training for longer periods of time explicitly point out that their workplace conflicts are more recent (reflective of changing culture). Also code when participants describe generational differences for women surgeons (how expectations have changed).
Surgical Culture: Statements regarding the nature of surgery (known to be aggressive, assertive, etc.). Include comments regarding role that specialty plays in workplace conflict and/or culture in general.
Intersectionality: Explicit comments regarding how other factors (such as race, place in hierarchy) factor into perceptions on workplace conflict. Also code when examples given seem to involve other factors (surgeon accused of racism, classism, etc.).
Impacts of Modeling/Leadership on Culture: Explicit statements regarding the role that leadership has on environment (number of women in leadership roles, business as usual, etc.). Also include examples of impacts from training.
Reflections on Larger Society: Statements regarding how things happening in workplace are not in a vacuum but rather reflect broader societal issues.
Reflections on Personal Life: Statements on how being a surgeon has impacted personal life (role as a mother, wife, friend, etc.).
Innate Personality: Examples of participants who state that a certain personality type tends to avoid conflict (more communicative, more nurturing, etc.).

© 2020 Dossett LA et al. *JAMA Network Open.*

Material Culture: Statements regarding physical barriers or obstacles that have a gendered component as it relates to the physical space (surgical instruments, stepstools, use of white coat to signal position, etc.).
Ideas for Moving Forward: Statements or insight into how to address issues of gender inequity in the surgical environment (more women in leadership roles, implicit bias training, having allies, etc.).
Complaints Against Non-Physician Staff: Examples of surgeons making reports against non-physician staff. Include statements that reflect why reports against non-physician staff were not made.
How Event Could Have Been Handled: Statements regarding how the event could have been handled
Is it Gender?: Statements that reflect the difficulty in teasing out things that are related to gender versus things that are not
Informal Rules of Being a Woman: Statements regarding the informal rules of being a woman surgeon (advice from senior women, friendliness, etc.)
Social Capital: Social groups that include interpersonal relationships, shared sense of identity, shared understanding, etc. (E.g. women surgeons who meet in community once a month). Also consider coding for 'Ideas for Moving Forward'
Establishing Boundaries: Idea that non-physician staff often expect women surgeons to treat them more like a friend than an employer and need to establish boundaries (be nice but not too nice)
Impacts to Reputation: Explicit comments regarding impacts or concerns regarding impacts to reputation. Might overlap with career trajectory.
Relationship Management: Explicit statements regarding how participants manage own behavior/feelings in order to 'control' responses of other team members. Might also be considered emotional intelligence.
Female to Female Conflict: Explicit statements about women being resistant to taking orders from women in higher positions.
Exemplary Quotes: Code for exemplary quotes**.**
Parking Lot: Areas of future inquiry.

Other Ideas

Punitive culture – might just fit into surgical culture
Nursing culture vs surgical culture
Resilience – examples of things being resolved

© 2020 Dossett LA et al. *JAMA Network Open.*
